# The Metamorphosing Professor: Adapting Teaching to Fulfill the Promise of Biology Education

**DOI:** 10.1093/icb/icac149

**Published:** 2022-09-29

**Authors:** Marcos E García-Ojeda, Michele K Nishiguchi

**Affiliations:** Department of Molecular and Cell Biology, University of California, 5200 Lake Rd, Merced, CA 95343, USA; Department of Molecular and Cell Biology, University of California, 5200 Lake Rd, Merced, CA 95343, USA

## Abstract

Teaching students at all levels of education has undergone extensive changes, particularly in the past decade. Our present student population has transformed dramatically in the 21st century due to the changing demographics of the nation, an increasing use of technology both inside and outside the classroom, along with an expectation to have information instantaneously available to peruse and utilize as a source of material. Today’s instructors also need to adapt to these changes by assessing how well students are learning new concepts, as well as how much material students retain for future coursework. Here, we explore the recent history of science education, and the progress that has been made to overcome multiple learning obstacles, particularly relevant to PEERs (persons excluded because of their ethnicity or race) in STEM (science, technology, engineering, and mathematics). We hope to provide insight into how educators are restructuring the way they design their teaching portfolios to provide better outcomes for the students of today’s educational system.

## Introduction

Spring quarter at the University of California at Davis, where bicycles are freely wheeling their way around campus and students are eager to finish their last quarter before summer break. It was 1984, and the biochemistry department always offered their Enzyme Kinetics course MWF at 8:00 AM. As a biochemistry major (MKN), this was one of the requirements to graduate; thus, everyone that was in that major was jammed into the classroom those early spring mornings. Although class was slated to commence at 8:00 AM, students usually arrived by 7:45 AM since the entire sliding chalkboard was already covered with equations in colored chalk that we needed to write down as part of the lecture material. At exactly 8:00 AM, the professor would have us turn in our homework, and commence with the material that was already on the board. If you arrived late, you would not have enough time to write all the notes on that board, let alone listen to what the professor was saying about the material. Ten minutes later, the sliding chalkboards would be moved, exposing a new set of equations (more writing), or it would be erased so that more material would be presented in this format. Write, listen, memorize, and regurgitate. One-way content dissemination and expected memorization—passive learning at its best instead of actively engaging the students.

What I can recall from that Biochemistry course many years later (besides going through numerous four-colored BIC^®^ pens) was how stressed I was about getting all the information written down in such a short amount of time, without having any recollection of whether I really understood what those equations meant or the relevance of a Michaelis–Menten constant. It was all about memorizing information and then reiterating it for the exam. Was this an ideal form of learning enzyme kinetics, or was it just the way that traditional classroom teaching (Guskey 2007) had been executed through the long-standing tradition of lecturing as much information to the class and expecting students to remember all that was delivered? Assessment, particularly regarding instructor performance, was not even considered. Students were solely evaluated on exam and homework scores, without assessment on critical thinking skills, communication, or retainment of the material (Gardner et al. 1989; Coil et al. 2010). This type of knowledge transfer also marginalized students who had difficulty learning, particularly those who come from different educational backgrounds and cultures (Barr et al. 2008). Students not only needed the basic foundational literacies (scientific, computational, financial, cultural, and civic literacies) that are taught from their first day in school, but also the competencies of how students approach complex problems in a changing environment (Coil et al. 2010; Gual and Dadlani 2020). Today, instructors are limited in the amount of time it takes to learn how to teach students in this manner (Coil et al. 2010). This is especially imperative, given the speed at which technology is advancing, and how the future generation of scientists will be able to approach and tackle complex problems. If we are to transform the way we teach students, we must give them toolkits to be self-directed, collaborative, and open to different ideas (attitude), better preparing them to tackle future questions that are not even evident to us today (OECD 2019).

## Our students are changing but our pedagogical approaches are not mirroring that change

Students in our classrooms look fundamentally different from the classmates we had during our undergraduate studies. A report from the National Commission on Asian American and Pacific Islander Research in Education (CARE), stated that minority students in the classroom increased from 10% in 1950 to 25% by 2010 (National Commission on Asian American and Pacific Islander Research in Education (CARE) 2011). As this demographic trend continues, PEERs (Persons Excluded because of their Ethnicity or Race) (Asai 2020), particularly Hispanics and Asian American/Pacific Islanders (AAPI), will reach majority status by 2050 (Anderson 2003; Teranishi and Kim 2017). Furthermore, more women are attending and graduating from college than men (Conger 2015; Syed and Johnson 2021). Additionally, the presence of other diverse group of students, such as first-generation, LGBTQIA+, disabled, and neurodivergent students (i.e., students living with attention-deficit/hyperactive disorder (ADHD), dyslexia or autism, among others) can remain hidden in our classrooms, making it difficult to address learning inequities among our students (Eddy and Hogan 2014; Lefler et al. 2016; Cooper et al. 2019; Carroll et al. 2022; Nardo et al. 2022). Thus, our changing demographic of college students brings both challenges and a much-needed diversity to American college classrooms than ever before (Sharon and Baram-Tsabari 2020).

Presently, students also have different generational experiences than us. Often referred to as “digital learners” (Evans and Robertson 2020) or iGens (Twenge 2017), today’s students grew up with smartphone technology, the internet, and social media, representing the newest generation of digitally engulfed people. This exposure to, and dependence on, technology comes with challenges, such as reduced capacity to multitask as well as the presence of constant internet distractors (Twenge 2017; Feng et al. 2019).

The diversity observed in American college classrooms is not mirrored by the faculty in those classrooms. The STEM (science, technology, engineering, and mathematics) professoriate remains primarily white (Miriti 2020) and male (Griffith and Dasgupta 2018), often leading PEERs, women, and LGBTQIA + students to experience lack of interpersonal relationships with faculty, which contributes to low sense of belonging in STEM fields (Rainey et al. 2018; Cooper et al. 2020). Nationwide, faculty identified as PEERs account for 20% of the professors working at degree-granting universities. In STEM, PEER faculty embody 9% of the professorate (Miriti 2020). This raises the question: what pedagogical approaches are faculty using to teach these diverse and technologically savvy students? Primarily, lecture (Stains et al. 2018; Alkhouri et al. 2021; Denaro et al. 2022) is now being replaced by less traditional methods, such as active learning in team-based or flipped courses, as well as CUREs (Course based Undergraduate Research Experiences), having a large impact on how students learn in this modern classroom.

## Biology education, then and now

Much of what has been the beginnings of self-directed or active learning was initially founded in physics in the mid- 1970s (Meltzer and Thornton 2012). Students oftentimes were having difficulties learning concepts specific for a physics course, as well as resolving their own beliefs and learning behaviors as to what they needed to do to understand the material. Many physics professors realized that students were much better at understanding concepts when applying those ideas in laboratory courses that were aligned with a particular lecture or course material. These laboratory exercises aimed to generate a better understanding of course content, and included laboratory experiments, mathematical modeling, or simulations (Meltzer and Thornton 2012). When students were engaged in their own learning, they demonstrated the ability to comprehend these broader ideas, given that the content was presented in a more approachable and accessible manner.

Biology educators were not far behind in adopting evidence-based, hands-on active learning (Council 2010). Students got the opportunity to engage in their own learning, learn from their peers, and utilize team-based work to solve complex problems. This team-based approach encouraged students to reflect on how they were able to problem solve, both independently and when they worked together (Michael 1998). Peer-led workshops represent another mechanism for students to gain knowledge by teaching material that they previously learned (peer-leaders) to groups of students (peers) that are engaged in an activity and work together to tackle a difficult concept (Preszler 2009). Studies have shown that peer-led teaching, through mechanisms such as workshops, increases competency mastery and success in biology, particularly in PEERs as well as women, although all groups benefit from this type of learning structure (Preszler 2009).

## What is active learning?

Active learning implies that students are not passively receiving information (Prince 2004; Petress 2008). At its core, active learning requires students to be cognitively and meaningfully engaged with course material, as opposed to simply receiving it (Chi and Wylie 2014). In active learning, students and instructors work as partners in the learning process: the instructor scaffolds activities that present course material, acting as a facilitator of the learning experience. On the other hand, students engage with these learning activities, actively thinking about the material and what they are doing with it.

There is overwhelming evidence that active learning is superior to lecturing in student’s outcomes (Prince 2004). The most compelling study is a metanalysis by Freeman and colleagues that evaluated 225 studies of STEM classes (Freeman et al. 2014). They showed that the mean failure rate in courses taught using active learning is 21.8%, as opposed to lecture courses, which had a mean failure rate of 33.8%. The study also showed that active learning increased performance on tests, while lecturing increased failure rates by 55%. These benefits happened across STEM disciplines, irrespective of class size, course type, and course level. When discussing an odds ratio of 1.95 for the risk of failing a STEM course, they stated that. . .

If the experiments analyzed here had been conducted as randomized controlled trials of medical interventions, they may have been stopped for benefit—meaning that enrolling patients in the control condition might be discontinued because the treatment being tested was clearly more beneficial. (Freeman et al. 2014)

This is important, as studies also showed that active learning disproportionally benefits PEER and women students (Lorenzo et al. 2006; Haak et al. 2011).

Additionally, multiple studies demonstrate that faculty are still using teacher-centric lectures as their primary means of content delivery (Stains et al. 2018; Kranzfelder et al. 2020; Alkhouri et al. 2021). For example, Stains and colleagues used COPUS, the Classroom Observation Protocol for Undergraduate STEM (Smith et al. 2013), to evaluate instructor and student classroom behaviors in 2008 STEM courses at 24 PhD-granting institutions. They observed that most STEM faculty use didactic practices (primarily lecture), even when compelling evidence of lecture’s limited impact in student’s performance is abundant (Freeman et al. 2014). In Biology classrooms, about half of faculty evaluated use didactic practices, while <25% of biology faculty use student-centered practices (Stains et al. 2018). However, the use of student-centered, active learning pedagogies is slowly increasing (Haak et al. 2011; Stains et al. 2018), having a greater impact on student learning.

COPUS quantitates the types of behaviors instructors and students engage in the classroom but does not provide insights into the quality of these interactions (Kranzfelder et al. 2020). That level of granularity can be determined by evaluating instructor discourse. To achieve this, Kranzfelder and colleagues created the Classroom Discourse Observation Protocol (CDOP; [Kranzfelder et al. 2019]), which allowed them to examine the type of teacher discourse moves (TDM) used by biology faculty in their teaching. TDMs are conversational approaches used by teachers that support student understanding of content material (Kranzfelder et al. 2020). These differ from “instructor talk,” which is non-content communications (Seidel et al. 2015). TDMs promote learning by engaging students in a deeper understanding of the content material (Alkhouri et al. 2021). Their work found that most instructors, even those identified by COPUS as using student-centered practices in their classrooms, still use authoritative, non-interactive discourse moves, which are teacher-centered discourse practices that dominate classroom conversations. Interestingly, teaching faculty, whose primary responsibility involves teaching as opposed to research, use more student-centered, dialogic, and interactive TDMs (Alkhouri et al. 2021). Therefore, faculty incorporating active learning activities in their classrooms must remain cognizant of also using student-centered dialogic, interactive TDMs in the delivery of their content.

Another student-centered pedagogy is the flipped (or inverted) classroom. In the flipped classroom, students are exposed to content material prior to attending class, via specific assignments or video activities that traditionally present lecture material (Brewer and Movahedazarhouligh 2018; Styers et al. 2018; Shay et al. 2020). In class, the instructor guides the students through discussions, additional activities, or working through follow-up problems (Akçayır and Akçayır 2018). Students are thus in charge of their own learning process and are responsible for knowing some of the content prior to attending the next class session (Bergmann and Sams 2012). Instructors are then able to engage students more directly and can focus on challenging concepts that students might have struggled with rather than discover their lack of understanding through summative assessments. Multiple studies have shown that using a flipped classroom model has several advantages for student learning, where the development of critical thinking skills, ability to solve problems, and team-based learning enhance the educational experience (Missildine et al. 2013; Brewer and Movahedazarhouligh 2018). Although these advantages are an improvement from previous best practices of teaching in STEM, there are still challenges that exist that hinder use of flipped courses. These include time for redesigning courses, student behaviors that hinder their ability to manage time outside of the classroom, student buy-in, and how individual students independently learn the material prior to attending class (Betihavas et al. 2016; Akçayır and Akçayır 2018). Additionally, the variable use and availability of technology (instructional videos, computer skills, and Wi-Fi access), work or personal obligations, as well as less effective ways of communicating (e.g., feedback from homework) have hindered both instructors and students from successfully navigating a completely flipped classroom.

The use of case studies in team-based learning has also been an effective way to engage students in their course work (Smith and Murphy 1998; Leupen 2020). Case studies provide teams of students the opportunity to investigate a complex problem and use a variety of resources that may be of personal interest to them (e.g., diseases or the environment, Silverman and Welty 1990). Depending on the level of the class, case studies can be quite simple (identifying a microbe), or complex (designing a plasmid for a novel capability), might include data analysis (analytical skills), additional reading (researching the disease), and a report (communication skills, both oral and written). Students work in teams, and depending on time and the case’s complexity, the instructor can integrate all the teams in discussions, such that a larger and more complex idea is revealed when the class reconvenes at the end of the study. For example, we use the environmental problem of paper waste pollution as a “hook” to interest students in tackling such a problem via their knowledge in microbiology (Shay et al. 2020). Students then gain knowledge about the microbiological and biochemical processes involved in cellulose breakdown, including protein secretion, nutrient uptake, and metabolism. Case studies such as this allow students to integrate various topics from the course, rather than obtain this knowledge in a topic-bound manner, and provide a solid foundation for students to build their knowledge base (Thompson et al. 2020). Fortunately, case study repositories, such as the National Center for Case Study Teaching (https://www.nsta.org/case-studies) and peer-reviewed journals like CourseSource (https://qubeshub.org/community/groups/coursesource/), eliminate the need for instructors to develop and curate case studies, lowering the barriers to their implementation. Additionally, case study databases have expanded into other STEM disciplines, allowing instructors to choose from various levels of science and interdisciplinary studies that converge many different facets of STEM.

**Fig. 1:**
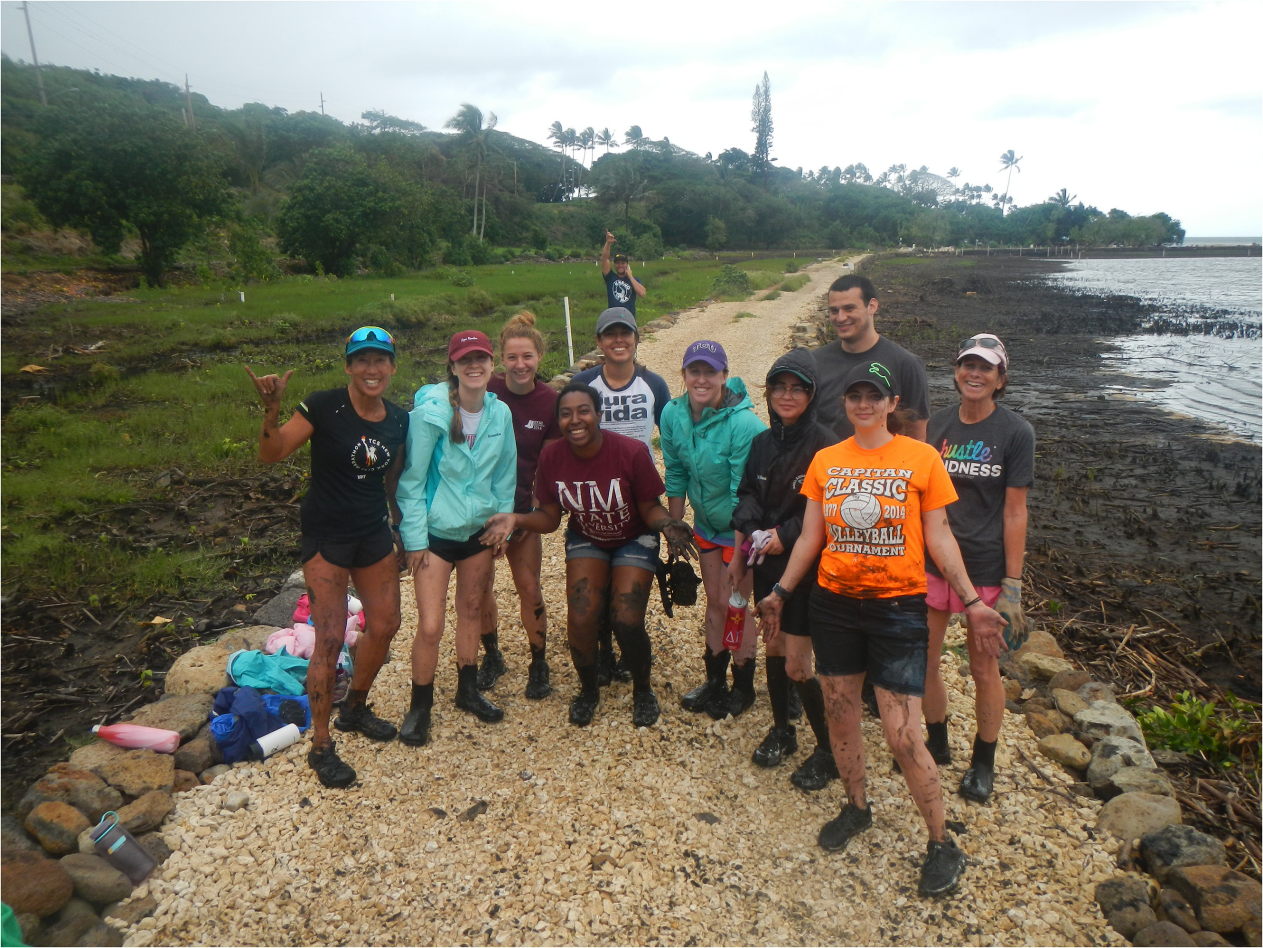
Field courses such as this corals and climate change class provide experiential learning that develops critical thinking skills to solve real-world problems. Here, students volunteer at Paepae o He'eia Fishpond in O'ahu, Hawaii.

A recent development at many universities is the creation and implementation of Course based Undergraduate Research Experiences (CUREs). These courses have been shown to increase the outcomes that are specified in Vision and Change (Brewer and Smith 2011), and provide innovative research experiences to students from a diverse student body. Students in CURE courses develop skills in critical thinking, experimental design, and quantitative analysis, by providing a chosen topic that helps develop and then implement those skills in a structured approach (Oufiero 2019). CUREs have also been shown to not only engage our STEM students in their learning abilities but also provide a more inclusive environment for our diverse student body to be exposed to research (Fig. 1).

One major issue that instructors implementing student-centered pedagogies face is the amount of time spent to not only incorporate new course design, but also the assessment and grading time that case study-based courses require. Instructors can use alternative strategies such as de-emphasizing high-stakes assessments (exams, large term papers [Odom et al. 2021]), and increasing low-to-medium-stakes assignments or using specifications (mastery) grading. This provides a clearer direction to the relevance of assignments, rubrics used for assessment, and engages the students in their educational path (Nilson 2015). Similarly, universal design for learning in course development allows accessible and transparent assignments, and subsequently saves the instructor’s time. This also paves the way for instructors to be more open to developing or changing their courses without having the additional class workload that is normally assigned during the academic year (Rose and Meyer 2002).

## A changing future for STEM education—implementing change based on inclusivity, equity, and diversification among our future scholars

The future of STEM education comprises a variety of avenues that direct the way educators are teaching courses to implement change in our way of thinking about pedagogy. Most of the focus has been on lower-division, first-year undergraduate courses, where retention is low and attrition high (Haak et al. 2011). Since this is the starting point for universities to begin tracking and assessing students and student success, most of the financial investments have been placed on these early career scholars (supplemental courses, tutoring and advising centers, and living-learning communities). Yet, there are still inadequacies in transforming our efforts from lower-division, active learning intensive courses to upper-division, major-specific classes. There is also a lack of convenient and consistent (within and between universities) software that allows universities to track students, particularly when moving between majors or transferring from a different university for their upper-division coursework. An ideal system would be to track each student individually, such that their past performance in each class can guide instructors on how best they can achieve the next set of learning outcomes that will benefit future coursework. This is a monumental task yet needs to be resolved for society to keep up the pace of newer technologies and rapidly acquired information in STEM disciplines. In addition, multiple studies show that tenured and tenure-track faculty are more impactful for undergraduates than contingent (adjunct) faculty (Ehrenberg and Zhang 2005; Umbach 2007; Jaeger and Eagan 2011). Given that most universities still evaluate their faculty based on their research more than their teaching (Vogt 2008), our tenure-track faculty are assigned smaller, upper division courses that require less administrative time, and contingent faculty teach the larger, lower division courses that require more effort due to the sheer number of student contact hours.

Besides universities, scientific societies have an obligation to provide guidance for our future teachers and scholars (Thompson et al. 2020). Multiple societies now offer sessions on science education at their annual meetings, promoting best practices on teaching science in the classroom. Pedagogical workshops are also being offered during annual meetings and can provide new, evidence-based ways of teaching science in the classroom, both remotely and in person. The education committees of some societies focus their energy on including plenary speakers who have led the way of instigating new active learning techniques and provide a viewpoint that is constantly changing in today’s curriculum [e.g., the Moore lecture at the Society for Integrative and Comparative Biology (SICB) annual meeting]. SICB’s education council also recognizes outstanding contributions to science teaching and innovation by awarding a member with the M. Patricia Morse award, which was named after one of their members who dedicated her career to advancing the way educators teach science. This is particularly important in today’s society, where PEER students are highly impacted by their ability to participate in their own learning—a major stepping-stone in active learning (Thompson et al. 2020). Training our present instructors to think forward on how our next generation of students will combine multiple concepts to tackle complex problems is probably the greatest challenge we have at this moment.

Scientific societies also play an important role in guiding the content of their discipline courses. For example, the education committees of some societies have published curricular recommendations to inform course content. This includes the American Society for Microbiology (ASM) recommendations for microbiology courses (Merkel 2012), and the American Association of Immunologists (AAI) recommendations for immunology classes (Porter et al. 2021). The ASM curricular recommendations were modeled after the core concepts and competencies outlined in the AAAS/NSF Vision and Change Report (Brewer and Smith 2011). This report emphasizes the implementation of five core concepts for biological literacy in biology courses: ((i) evolution; (ii) structure and function; (iii) information flow, exchange, and storage; (iv) pathways of transformation of energy and matter ; and (v) systems). It also recommends that instructors shift the focus of their instruction from faculty-centered pedagogies like lecturing to student-centered approaches using active learning. The report also advises staying away from presenting all the facts about a subject (the dreaded content monster) to articulating clear learning outcomes that are easily assessable.

Scientific societies have always had an underlying obligation to share scientific knowledge within a specific subject, and now it is more evident that the knowledge needs to be shared across disciplinary boundaries. This also parallels the need to integrate and diversify membership within the society, since inclusion from various groups can greatly impact the way science solves complex problems. Recently, many scientific societies have been tracking the demographics of their society, with particular focus on PEERs and their attendance and participation in the society’s activities (meetings, workshops, and leadership [Segura-Totten et al. 2021; Burnett et al. 2022]). Data have shown that financial support greatly increases the ability of PEER scholars to attend and present their data at these national meetings (Wilga et al. 2017; Segarra et al. 2020; Odom et al. 2021). Additionally, PEER members who actively participate in STEM events centered around their achievements and successes promote camaraderie, a sense of belonging, and a broader view of scientific knowledge. Although societies have promoted PEER involvement through the addition of broadening participation committees, financial support, workshops, and award/recognition ceremonies, there is still a lack of representation within the upper levels of leadership, as well as continual membership throughout their career trajectory (Wilga et al. 2017). Societies are just beginning to collect data on their membership demographics and are utilizing this information to be better informed and focus their attention on where the loss of PEER participation occurs (Wilton et al. 2022). This trend parallels what our universities are trying to accomplish, with hopes to provide a more inclusive and diverse set of scholars who are better equipped to tackle future problems.

**Fig. 2:**
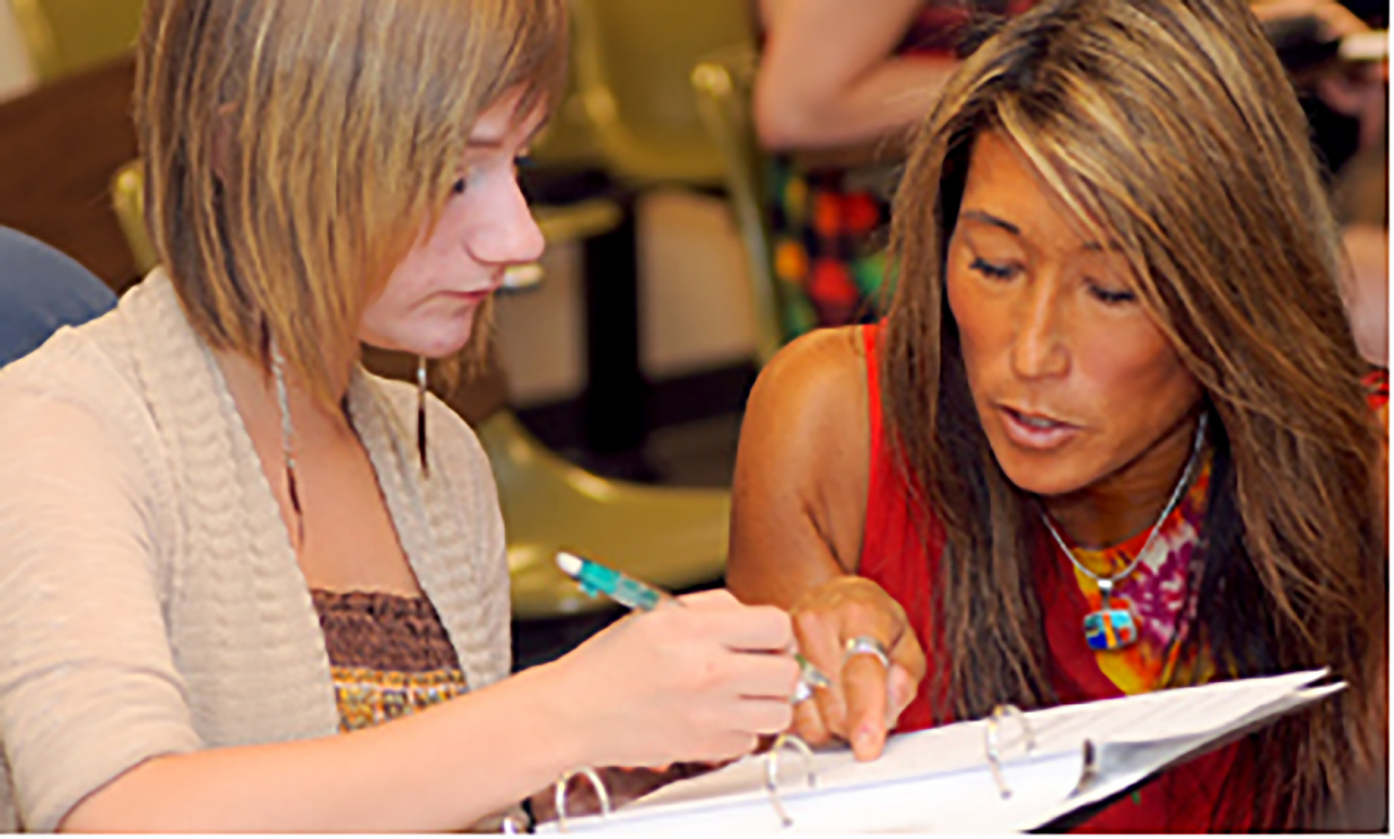
Nishiguchi uses multiple active learning techniques to engage students in her microbial genetics course.

Finally, the study of science education is now becoming more inclusive, with STEM departments hiring faculty whose research is in science education. This trend has not only provided the necessary input from scholars who study how science can be taught effectively, but also provided an “expert” who can provide the guidance needed for STEM departments to reform their curriculum, as well as better assess how our present-day students are learning and retaining information (Hodson 2003; Fig. 2). If we can mirror similar demographics in our future faculty that match our student populations and change the way we approach how instruction is delivered to this “iGen” group of students, we will be better prepared as a society overall to tackle new challenges and creatively design innovative mechanisms to embark on the future of STEM research.

Finally, we end this opinion paper with a recent example from one of the authors courses (MGO). Fast forward to the fall 2022 semester at the University of California, Merced, where microbiology students are working in small groups to understand the structure of the cell wall of *Bacteria*. Each group is divided into two pods, and each pod is responsible for illustrating either the Gram-positive or Gram-negative cell wall. Initially, each student works independently in their drawing, using the slides from the video lecture they watched before attending class as reference. Eventually, members of each pod compare drawings, browsing their textbook or the internet to get in-depth information about the structure of the peptidoglycan layer. As a pod, students use their collective knowledge to illustrate the cell wall of their respective bacteria and fix mistakes. My co-instructor and I (MGO) visit each group to clarify points, provide feedback, and answer questions: “You nicely illustrated the glycosidic bond between the sugar moieties in peptidoglycan, but what type of bond is it? Is this type of bond also found in the pseudopeptidoglycan in *Archaea*?” The pods reconvene and each presents their final drawing to the other pod, the whole group discussing the details of the cell wall for both types of *Bacteria*. I (MGO) reconvene the class and let students describe the major differences between the cell wall of these microorganisms, providing them with time to modify their drawings one last time before turning them in as homework. The lesson is scaffolded so that students build upon previously presented material, allowing them to showcase what they learned, while flushing out misconceptions. Engage, explore, explain, elaborate, evaluate; student-centered content dissemination — active learning at its best instead.

## Funding

This work was supported by National Aeronautics and Space Administration Exobiology grant #EXO80NSSC21K0256 to M.K.N. and the School of Natural Sciences at UC Merced.

## Data Availability

No data was produced or analyzed for this manuscript.
